# Do Vaccines Need a Gender Perspective? Influenza Says Yes!

**DOI:** 10.3389/fimmu.2021.715688

**Published:** 2021-07-05

**Authors:** Laura Sánchez-de Prada, Raúl Ortiz de Lejarazu-Leonardo, Javier Castrodeza-Sanz, Eduardo Tamayo-Gómez, José María Eiros-Bouza, Iván Sanz-Muñoz

**Affiliations:** ^1^ Department of Microbiology, Hospital Clínico Universitario de Valladolid, Valladolid, Spain; ^2^ National Influenza Center of Valladolid, Hospital Clínico Universitario de Valladolid, Valladolid, Spain; ^3^ Department of Preventive Medicine and Public Health, Hospital Clínico Universitario de Valladolid, Valladolid, Spain; ^4^ Department of Anesthesia, Critical Care and Pain Medicine, Hospital Clínico Universitario de Valladolid, Valladolid, Spain; ^5^ Department of Microbiology, Hospital Universitario Río Hortega, Valladolid, Spain

**Keywords:** influenza, sex differences, influenza vaccine, sexual dimorphism, elderly

## Abstract

**Background:**

Sex differences in immune responses are well known. However, the humoral response in males and females in the case of influenza vaccination is yet to be characterized since studies have shown uneven results.

**Methods:**

A retrospective study was conducted in 2,243 individuals (46.9% males) divided by age (15–64 and ≥65 years old). A serological analysis was performed by hemagglutination inhibition assay (HI) just before and 28 days after annual vaccination against seasonal influenza viruses in people vaccinated during the 2006–2018 seasons. A comparison of the humoral responses against influenza A and B viruses contained in the vaccine, between male and female individuals in young adults and elderly was conducted.

**Results:**

Significative higher humoral response against classical influenza A (H1N1), A(H1N1)pdm09 subtype and B/Victoria lineage in terms of seroconversion rate were found in elderly women. No significant differences were found in the case of A(H3N2) subtype.

**Conclusions:**

Elderly women seem to display a greater humoral response against classical A(H1N1), pandemic A(H1N1)pmd09 and B/Victoria lineage than elderly men. Sex dimorphism does not affect young adults.

## Introduction

In many infectious diseases, gender is a factor that usually goes unnoticed and undervalued. However, COVID-19 has taught us that, sometimes, there are subtleties in morbidity and mortality differences regarding this particular topic (1) that need to be studied more closely. Mechanisms involved in these sex differences are complex and include immunological, hormonal, behavioral, and genetic factors, among others (2). As an example, women typically produce more intense innate and adaptative responses than men, which can be beneficial to rapidly reduce viral load, but harmful in the case of autoimmune diseases (3).

In the case of influenza, epidemics and pandemics are usually responsible for higher morbidity and mortality rates in women. According to the report by the WHO in 2010, pandemic influenza causes more severe and more deadly cases in women, especially in the youngest (4). In the recent influenza pandemic caused by A(H1N1)pdm09 subtype in 2009, young women (15–49 years old) were the group with the highest rates of hospitalization in the USA, Canada, and Australia (5–7). However, other studies have shown that the impact in terms of morbidity and mortality caused by influenza is higher in men in both extremes of life, under 20 and over 80 years old (8).

Some authors have also reported the existence of gender differences in influenza vaccination. Women typically suffered more frequently from local and systemic side effects (9). However, the antibody induction in women is usually higher than in men after vaccination (10). Some authors suggest that half of a dose of an influenza vaccine is able to produce the same humoral response in a woman as a complete dose of the same vaccine in a man (11).

Given the interest in understanding the details of humoral response against influenza vaccines, the object of this study was to analyze the differences in humoral responses against influenza A and B viruses in a vaccinated human cohort by gender and age, through 13 consecutive influenza vaccine campaigns.

## Materials and Methods

### Patient Recruitment and Population Characteristics

A retrospective observational study was designed for 2,243 recruited healthy individuals (1,052 men and 1,191 women) from 2006 to 2018. These subjects were recruited yearly by the clinicians of the Influenza Sentinel Surveillance Network of Castile and Leon (Spain) (ISSNCyL) during their medical visit for influenza vaccination. Pre and post-vaccination serum samples were obtained through the Influenza Vaccine Campaigns (IVCs) between 2006 and 2018. Pre-vaccination sera were obtained right before influenza vaccination, and post-vaccination samples at least 28 days after vaccination to ensure a correct immunization. Analyses of serum samples against the Classical A(H1N1) subtype were performed from season 2006 until 2011 and against the pandemic A(H1N1)pdm09 subtype from 2010 till 2018. Analyses against A(H3N2) subtype and influenza B lineages were performed in all seasons, except during 2015 till 2017 for A(H3N2) subtype; during these seasons the assays could not be performed due to deficiencies in the agglutinant power of hen erythrocytes caused by the antigenic changes in the virus (12, 13). The serum samples were stored at −20°C before until their analysis at the National Influenza Centre of Valladolid (Spain). The delivered seasonal influenza vaccines included the A and B influenza strains recommended by the World Health Organization (WHO) for the Northern Hemisphere for each IVC (14). In all seasons Trivalent Influenza Vaccine (TIV) was used except for 2018 where Quadrivalent Influenza Vaccine (QIV) was introduced as a split virus vaccine but the adjuvanted vaccine was still trivalent. The data of the type of vaccine used are detailed in **Table 1**.

**Table 1:** Details of the different vaccines used during each IVC following the WHO recommendations divided by gender and age.

	15–64 years old						≥65 years old					
Type	Split-virus		Adjuvanted		Not Defined		Split-virus		Adjuvanted		Not Defined	
IVC	Men	Women	Men	Women	Men	Women	Men	Women	Men	Women	Men	Women
2006–2007	11	14	–	–	1	5	37	25	10	20	3	3
2007–2008	20	20	–	–	–	–	30	27	15	39	–	–
2008–2009	19	11	2	0	1	0	17	29	31	61	5	2
2009–2010	20	12	–	–	10	8	26	22	27	36	19	22
2010–2011	17	19	–	–	3	2	16	16	57	69	2	1
2011–2012	22	18	–	–	–	0	21	15	30	46	8	10
2012–2013	22	16	2	0	2	1	41	52	20	20	4	3
2013–2014	18	25	2	1	–	–	12	20	39	54	8	14
2014–2015	21	23	–	–	–	–	10	6	53	55	1	0
2015–2016	17	31	0	1	0	1	8	4	47	58	–	–
2016–2017	12	21	2	1	–	–	0	1	57	69	–	–
2017–2018	22	24	1	0	–	–	0	1	64	56	0	1
2018–2019	17	26	2	2	–	–	4	0	63	52	–	–

**Figure 1:**
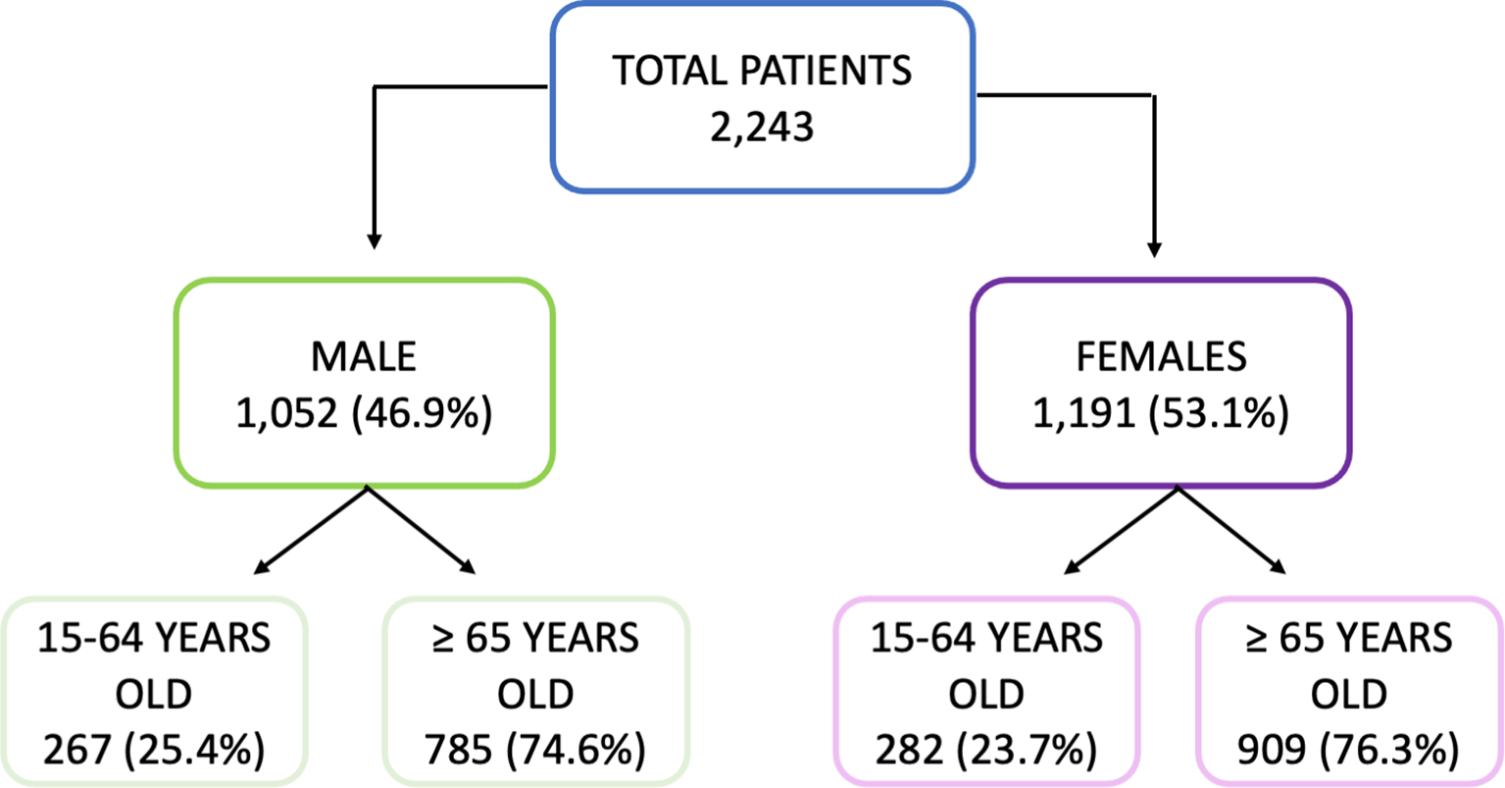
Diagram of the cohort selection.

Of the total number of individuals recruited for this study 1,052 (46.9%) were male; 267 (25.4%) were 15–64 years old and 785 (74.6%) were ≥65 years old. A total of 1,191 females were included of whom 282 (23.7%) and 909 (76.3%) were 15–4 and ≥65 years old, respectively. These constitute the four study groups. The median age of each group was: 57 (SD: 9.2) and 53 (SD: 11.5) years for men and women 15–64 years old, respectively; and 77 (SD: 7.6) and 80 (SD:8.9) years for men and women ≥65 years old, respectively. The median age was significantly higher in men for the adult cohort and significantly higher in women (Mann–Whitney, *p* < 0.05) for the elderly cohort (**Figure 1**). The selection of the age criteria was carried out based on the classical age groups used in influenza studies. All subjects gave their informed consent before inclusion in the study, and the recruitment of patients was performed following the Spanish Organic Law for Data Protection, patient’s rights and obligations for clinical documents (BOE n°298 of14 December 1999, Law 41/2002). This research was performed according to the Declaration of Helsinki and was yearly approved by the Ethics Committee of East-Valladolid health area under the code PI 21-2314.

### Hemagglutination Inhibition Assay

The hemagglutination inhibition assay (HI) was performed to detect and quantify the presence of anti-hemagglutinin antibodies (Abs) in pre- and post-vaccination sera. This analysis was conducted following the protocol recommended by WHO and the Influenza Surveillance Network for the surveillance of influenza viruses and vaccine efficacy (15). Before the HI, the serum samples were pretreated with RDE following the manufacturer’s instructions (Denka Seiken, Tokyo, Japan). Then, this mixture was diluted with 600 μl of PBS to reach a working solution concentration of 1/10.

Briefly, to perform HI, the virus was standardized to 4 hemagglutinin units (4 HU) and hen erythrocytes at 0.75% were employed. The Ab titer was defined as the highest dilution presenting complete hemagglutination inhibition. Pre- and post-vaccination titers were then included in a database. For this analysis, the A and B vaccine strains designed by WHO for each IVC were used. A PBS negative control and a viral-only positive control were employed in each plate. Additionally, to assess the presence of unspecific inhibitors, a serum control, which included only the serum sample without a virus, was used.

### Statistical Analysis

The results were analyzed by using the classical serological criteria of the European Medicament Agency (EMA) for the evaluation of serological response to influenza vaccines. Those criteria evaluate different parameters, such as the seroprotection rate (SPR) (percentage of individuals with antibody titers ≥1/40), seroconversion rate (SCR) (percentage of individuals showing at least a four-fold increase from pre-vaccination titers) and the geometric mean titers (GMTs) and their increase (GMTi). The GMTi was calculated by dividing the post-vaccine GMTs and pre-vaccine GMTs. Negative results in HI were assumed as half of the detection threshold (1/10). Seroconversion was defined as a titer increase of at least four-fold between pre- and post-vaccination sera (15–17). In addition, seroconversion in cases with negative titers in the pre-vaccination serum was only recorded as such when the post-vaccination serum reached a titer ≥1/40. Different statistical non-parametric tests were used, using SPSSV26 (IBM, Armonk, NY, USA) and GraphPad Prism V8 (GraphPad, San Diego, CA, USA) and taking statistical significance at the p-value <0.05.

## Results

### Humoral Protection Before Vaccination

Seroprotection and the presence of Abs before vaccination of the population studied were analyzed through a descriptive study of the pre-vaccination GMTs and SPR (**Table 2**). For classical A(H1N1) subtype, the highest SPR was found in men 15–64 years old (57.5%) and the lowest in women ≥65 years old (38.2%). For A(H1N1pdm09) subtype, the highest value was 73.1% in women 15–64 years old and the lowest was 64.7% in women ≥65 years old again. For A(H3N2) subtype, women 15–64 years old presented the lowest SPR (70.9%) and men ≥65 years old presented the highest SPR (73.8%). For B/Yamagata and B/Victoria lineages, the lowest SPR was found in women 15–64 years old (68.1%) and the highest SPR in men ≥65 years old for B/Yamagata lineage.

**Table 2:** Humoral HI response to seasonal flu vaccination by age and gender groups. Pre-vaccination values of the geometric mean titers (GMTs) and seroprotection rate (SPR).

Strain/Subtype	Pre-vaccine	Vaccinated cohorts			
		Men 15–64	Women 15–64	Men ≥ 65	Women ≥65
**A(H1N1)**	GMTs (CI 95%)	36.2 (27.8–47.1)	30.6 (23.4–40.2)	25.1(22.2–28.4)	22.7 (20.3–25.4)
	SPR (%)	57.5	42.9	43.7	38.2
**A(H1N1)pdm09**	GMTs (CI 95%)	75.3 (60.3–94.1)	90.0 (72.3–112.0)	55.2 (49.0–62.3)	49.3 (44.2–54.9)
	SPR (%)	72.1	73.1	66.4	64.7
**A(H3N2)**	GMTs (CI 95%)	60.9 (49.9–74.3)	66.6 (54.9–80.8)	72.7 (64.8–81.5)	74.0 (66.7–82.1)
	SPR (%)	71.2	70.9	73.8	73.4
**B/Yamagata **	GMTs (CI 95%)	123.7 (104.6–146.4)	119.4 (102.0–139.8)	117.6 (107.5–128.6)	122.1 (112.4–132.7)
**lineage**	SPR (%)	84.6	85.5	87.4	87.3
**B/Victoria**	GMTs (CI 95%)	80.8 (67.5–96.9)	63.7 (53.6–75.6)	103.5 (93.9–114.1)	97.4 (89.2–106.3)
**lineage**	SPR (%)	75.3	68.1	82.8	82.0

For the classical A(H1N1) subtype, the highest GMTs were found in men 15–64 years old (36.2, CI95%: 27.8–47.1) and the lowest in women ≥65 years old (22.7, CI95%: 20.3–25.4). For A(H1N1)pdm09 subtype, the highest value was 90.0 (CI95%: 72.3–112.0) in men 15–64 years old and the lowest again in women ≥65 years old (49.3, CI95%: 44.2–54.9). Women ≥65 years old presented the highest GMTs, and men 15–64 years old presented the lowest for A(H3N2) subtype, with values of 74.0 (CI95%: 66.7–82.1) and 60.9 (CI95%: 49.9–74.3) respectively. For lineages of type B virus, the highest GMTs were found in men 15–64 and ≥65 years old for B/Yamagata lineage (123.7, CI95%: 104.6–146.4) and B/Victoria lineage (103.5, CI95%: 93.9–114.1), respectively. Finally, the lowest GMT values were 117.6 (CI95%: 107.5–128.6) for B/Yamagata lineage in men ≥65 years old and 63.7 (CI95%: 53.6–75.6) for B/Victoria lineage in men 15–64 years old.

No significant differences were found for the pre-vaccination GMTs in adults (15–64 years old) and in the elderly (≥65 years old) between men and women for A subtypes nor for B lineages (Mann–Whitney, *p* < 0.05). Also, no significant differences were found in SPR for any group and influenza virus, but only for A(H1N1) classic subtype when comparing men and women 15–64 years old (χ^2^, *p* < 0.05).

### Humoral Response and Protection After Influenza Seasonal Vaccination

The response to vaccination was analyzed by four different parameters related with humoral response and seroprotection: SCR, post-vaccine GMTs, GMTi, and post-vaccine SPR. Those parameters are described in **Table 3**.

**Table 3:** Values of the seroconversion rate (SCR), geometric mean titers (GMTS), GMT increase (GMTi), and seroprotection rate (SPR) by HI against types and subtypes of influenza viruses after seasonal flu vaccination, by groups of age and gender analyzed.

Strain/Subtype	Pre-vaccine	Vaccinated cohorts				Significance (*p-value*)	
		Men 15–64	Women 15–64	Men ≥ 65	Women ≥65	Men *vs*. Women 15–64	Men *vs*. Women ≥65
**A(H1N1)**	SCR (%)	27.9	37.4	30.2	40.6	0.158	0.005*
	GMTi	2.2	3.2	2.3	2.9	0.123	0.055
	GMTs (CI95%)	80.5 (62.0–104.7)	99.0 (75.1–130.5)	58.0 (50.6–66.5)	66.5 (58.9–75.2)	0.306	0.117
	SPR (%)	77.9	81.3	68.8	74.5	0.554	0.147
**A(H1N1pdm09)**	SCR (%)	48.2	46.7	42.0	52.4	0.789	0.001*
	GMTi	3.6	3.6	3.0	3.7	0.579	0.000*
	GMTs (CI95%)	268.8 (227.3–318.0)	327.4 (283.3–378.4)	163.2 (146.8–181.3)	182.7 (166.2–200.8)	0.184	0.077
	SPR (%)	95.6	96.2	89.9	93.7	0.909	0.542
**A(H3N2)**	SCR (%)	39.6	45.3	44.7	47.1	0.250	0.399
	GMTi	3.4	3.5	3.2	3.3	0.983	0.175
	GMTs (CI95%)	204.5 (169.4–246.8)	232.8 (195.7–276.9)	233.7 (210.1–260.0)	247.5 (226.0–271.2)	0.667	0.286
	SPR (%)	92.0	95.0	93.3	94.9	0.402	0.865
**B/Yamagata**	SCR (%)	24.6	24.4	21.0	24.6	0.961	0.092
**lineage**	GMTi	2.0	2.1	1.7	1.9	0.987	0.108
	GMTs (CI95%)	246.2 (211.5–286.6)	247.2 (215.8–283.2)	205.1 (187.4–224.3)	227.7 (210.5–246.4)	0.983	0.158
	SPR (%)	95.9	96.8	93.9	95.5	0.805	0.980
**B/Victoria**	SCR (%)	26.6	28.3	18.9	23.2	0.664	0.039*
**lineage**	GMTi	2.3	2.3	1.9	1.9	0.317	0.243
	GMTs (CI95%)	183.1 (154.8–216.7)	146.5 (124.7–172.0)	193.1 (176.3–211.6)	187.5 (172.8–203.4)	0.053	0.557
	SPR (%)	95.9	96.8	93.9	95.5	0.805	0.649

P-values of the comparison between both genders for each age group. Significant differences (p<0.05) are marked with *.

**Figure 2:**
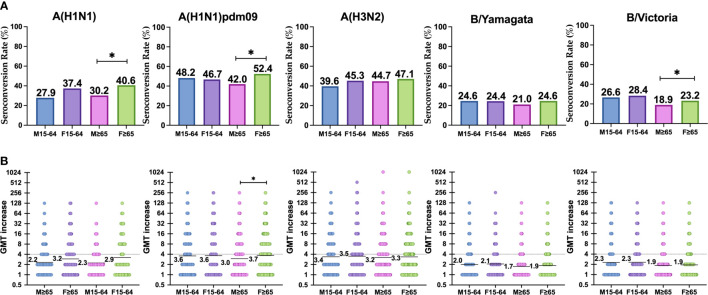
**(A)** Represents the reponse to vaccination in terms of SCR (four-fold-induction of GMTs) in each group against each subtype. **(B)** Represents the increase of GMTs after vaccination in each group against each subtype. Significant differences (p < 0.05) are marked with *.

The highest SCR achieved in adults was observed against the A(H1N1)pdm09 subtype in men (48.2%) (**Table 3**) and the lowest against B/Yamagata lineage in women (24.4%). In the case of the elderly, the highest SCR was achieved against the A(H1N1)pdm09 subtype (52.4%) in women and the lowest against B/Yamagata lineage (21.0%) in men. The SCR was significantly higher in elderly women than in men for the A(H1N1)pdm09 and classical A(H1N1) subtypes, and also for the B/Victoria lineage (Pearson χ^2^, *p* < 0.05) (**Table 3** and **Figure 2**). No other significant differences were found between both genders for any influenza virus in adults and in the elderly. The lowest SCR for A(H3N2) subtype was found in men 15–4 years old (39.6%) and the highest in females ≥65 years old.

The highest GMTi was observed in adults against A(H1N1)pdm09 subtype for both men and women (3.6), and the lowest against B/Yamagata lineage in men (2.0). In the elderly, the highest GMTi was observed against A(H1N1)pdm09 subtype in women (3.7), and the lowest against B/Yamagata lineage in men (1.7). The GMTi was significantly higher in elderly women than in men for the A(H1N1)pdm09 subtype (Mann–Whitney, *p* < 0.05) (**Table 3** and **Figure 2**). No other significant differences were found between both genders for any influenza virus in adults and in the elderly. GMTi was 3.4 and 3.5 in the younger population for males and females respectively, whereas for the elderly it was 3.2 and 3.3 for males and females respectively.

**Figure 3:**
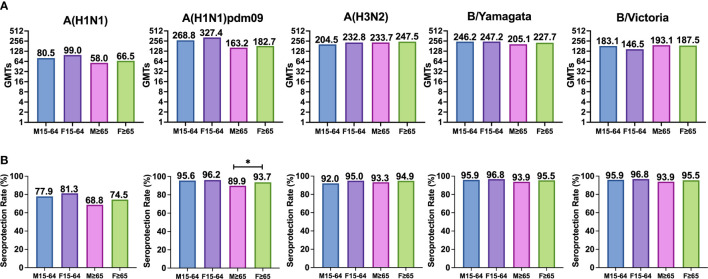
**(A)** Represents the GMTs and **(B)** represents the SPR achieved in both cases after vaccination in each group for each subtype. Significant differences (p<0.05) are marked with *.

In adults, the highest GMTs after vaccination were observed in women for A(H1N1)pdm09 and the lowest in men for classical A(H1N1) subtype. In the case of the elderly, the highest GMTs after vaccination were observed in women for A(H3N2) subtype and the lowest in men for classical A(H1N1) subtype. No significant differences were observed between both sexes against any virus for both age groups (Mann–Whitney, *p* < 0.05) (**Table 3** and **Figure 3**).

The highest post-vaccination SPR in adults was found in women for both B lineages reaching 96.8%, and the lowest was found in men for classical A(H1N1) subtype. In the elderly, the highest SPR was reached by women for both B type lineages (95.5%), and the lowest was achieved by men for classical A(H1N1) subtype. The analysis of SPR showed no significant differences in the comparison by gender in adults and in the elderly, except for the subtype A(H1N1)pdm09, which shows a significantly higher post-vaccination SPR in elderly women (93.7%) than in elderly men (89.9%). SPR for A(H3N2) subtype was above 90% in all groups with no differences between them (Pearson χ^2^, *p* < 0.05) (**Table 3** and **Figure 3**).

## Discussion

The role of gender in vaccine responses is an emergent field of interest. Most previous studies performed were not designed to find differences from a gender perspective. Due to this, most studies turn out to be inconclusive or showed no differences in immunogenicity as a recent review reported (18). In line with these findings, the results of our study suggest that before as well as after influenza vaccination, gender might not be a factor that affects either the basal seroprotection or the humoral response of an individual against influenza virus. However, we have detected some exceptions that are worthwhile mentioning, especially those related to increasing age and some viral subtypes, such as the ones belonging to A(H1).

Our data showed that before vaccination there are no significant differences by gender in the seroprotection analyzed in terms of GMT and SPR, when comparing gender in both adults and the elderly. This implies that men as well as women have, *a priori*, the same HI humoral protection against influenza virus in a basal status. Nevertheless, we observed a significantly higher SPR in young-adult men (57.7%) compared to women (42.9%) against classical A(H1N1) subtype. Similar findings were detected in a clinical trial performed with an inactivated influenza vaccine in healthy adults during two influenza seasons, finding a higher response against the classical A(H1N1) in men, but without any logical explanation for the data given by the authors (19).

From our point of view, a plausible explanation to this peculiarity in our data is that the median age of young-adult men was significantly higher than the median age of women of the same age range. This could denote that men would have been exposed to classical A(H1N1) subtype influenza viruses for longer periods during infancy and adulthood, such as those related to the influenza virus of 1918 that circulated before 1957 in addition to the subsequent exposures after its re-emergence in 1977. Meanwhile, in the case of women, the contact could have only happened with those after 1977, which could have limited the present SPR value. As a consequence of the lack of certainty with our current data, further research needs to be done with more restricted age ranges.

Conversely, when serological data was analyzed after vaccination, no significant differences were found between young-adult men and women, but we found these differences in the case of the elderly. In particular, the HI humoral response against the A(H1N1)pdm09 subtype was significantly higher in women ≥65 years old (20) compared to men of the same age group in terms of SCR and GMTi and also against classical A(H1N1) subtype and B/Victoria lineage in terms of SCR. This reflects that influenza vaccines engage in a more intense HI humoral response in elderly women compared to elderly men. Although there are studies that relate the role of testosterone with a lower response to influenza vaccination in both young and elderly individuals (10, 20), we found these differences only in elderly women. This higher response of elderly women to influenza vaccination could be derived from many reasons, such as immunological features, hormonal, behavioral and genetic factors; it is probable that not only one factor might be accountable for this matter, but the combination of all of them (1–4, 9–11).

Typically, humoral and cell-mediated responses due to vaccination and infection are more intense in women than in men (21). Also, women have shown higher basal levels of immunoglobulin as well as higher levels of antibodies in response to viral infections and vaccines than men (11, 22–24). By contrast, men have higher antibody responses towards bacterial vaccines such as tetanus, diphtheria, and pertussis (Td/Tdap) vaccines as well as the 7-valent and 23-valent pneumococcal vaccines (25–27). Previous serological studies had mainly focused the response to vaccination in the elderly, some of them resulting in a higher response in women with influenza vaccination (10, 28). Also, a clinical trial performed with influenza vaccination showed better performance of elderly women in achieving protective humoral responses compared to elderly men (29). However, in studies performed in younger cohorts, no differences were found (30).

Our results are consistent with other studies, which show, globally, that influenza vaccination does not account for sex in terms of efficacy of humoral response in young adults, but it does so in the case of the elderly. A study performed in older adults (50–74 years) goes beyond serological analysis identifying differences between male and female in PBMC fractions of CD4+ T cells and NK cells as well and potential mechanisms for sex effects in four gene clusters related to T, NK, and B cells whose expression levels differ after influenza vaccination (31).

The currently available data point that the adaptive immune response of elderly women may be preserved to a further extent than elderly men as aged males experience a more dramatic decrease in total numbers of T and B cells and a larger increase in senescent CD8+ T effector memory cells (31, 32), which should be considered for infectious diseases and their treatment and prevention. There is an insufficient number of studies comparing responses to viral vaccination between men and women to determine if these differences are caused by an actual higher response due to their slower decline in immunity.

In our study, the higher response in elderly women was observed although the median age of this group was significantly higher than in elderly men, and this presupposes a higher degree of immunosenescence. With a median age three years older, at those stages of life, immunosenescence is something to bear in mind and a better humoral response to influenza vaccination found in elderly women is something to be acknowledged. Some studies performed in animals have shown that, whereas both sexes were equally protected against lethal challenge with homologous virus after influenza vaccination, females were granted with greater protection against lethal challenge with heterosubtypic viruses (24, 33). Other studies have shown that antibody production by B cells, such as responses to an inactivated influenza vaccine, can be stimulated by estradiol at physiological concentrations in mice (34, 35). These facts should be further studied as a possible aid to better immunization achievements.

Furthermore, the fact that these higher values found in elderly women are only detected against A(H1N1)pdm09 subtype and not against other subtypes could be on account of some peculiarity of the mentioned subtype that we are not able to detect. In this regard, the data obtained in the study of the effect of 2009 pandemic showed higher hospitalization and incidence rates in women 15–49 years old (5–7). While it is not the age range where higher protection rates are found after vaccination, it is the same gender which might have some relation that we are not capable to elucidate with the study design.

Most of studies performed in the elderly to assess the efficacy and effectiveness of new vaccines do not recruit men and women equally, which can lead to sex bias that could be easily avoided by performing an adequate selection.

One of the weaknesses of our study is that we only evaluated the presence of antibodies, but we did not perform assays to analyze the cellular response. In this way, further studies are needed to understand the whole immunological response in terms of sex differences. On the other hand, because we included 13 IVC in this study, we could have missed some inter-seasonal differences derived from the different influenza strains recommended by WHO. Further studies should be performed with larger female groups as well as elderly groups in order to elucidate the factors that are linked to this sex dimorphism in response to vaccination.

## Conclusions

In summary, our study shows that, in general, gender is not a relevant factor for the humoral immunity against influenza, but, in certain cases and against specific influenza subtypes, there are differences between both genders. Before vaccination, men presented a higher SPR against the classical A(H1N1) subtype than women. But, after vaccination, the elderly women showed higher humoral responses than elderly men against A(H1N1)pdm09 subtype, despite their higher median age. Although the specific reasons for those differences were not studied in this work, it is probable that the reasons are multi-factorial and probably related with immunological features, hormonal, behavioral, and genetic factors. This information could be useful to focus vaccination in those persons that present poorer response to vaccines. In addition, the information obtained could be critical in situations where there is an increased demand and stock shortage, such as a pandemic. Our findings should be considered when recruiting older patients for future influenza vaccine efficacy studies, considering the differences observed by gender.

## Data Availability Statement

The raw data supporting the conclusions of this article will be made available by the authors, without undue reservation.

## Ethics Statement

The studies involving human participants were reviewed and approved by Ethics Committee of East Valladolid Area. Written informed consent to participate in this study was provided by the participants’ legal guardian/next of kin.

## Author Contributions

All authors contributed to the article and approved the submitted version. JE-B, RO, JC-S, and ET-G designed the methodology of the study. IS-M supervised the laboratory work. LS performed the formal analysis supervised by IS-M and JE-B. LS and IS-M prepared the original draft and the figures and JE-B, RO, JC-S, and ET-G revised the final manuscript.

## Funding

The research was funded by the Health Ministry of Castile and Leon, Spain.

## Conflict of Interest

The authors declare that the research was conducted in the absence of any commercial or financial relationships that could be construed as a potential conflict of interest.
